# Regular Cycles of Forward and Backward Signal Propagation in Prefrontal Cortex and in Consciousness

**DOI:** 10.3389/fnsys.2016.00097

**Published:** 2016-11-28

**Authors:** Paul J. Werbos, Joshua J. J. Davis

**Affiliations:** Department of Mathematical Sciences, Center for Large-Scale Optimization and Networks, University of MemphisMemphis, TN, USA

**Keywords:** backpropagation, synchronization, prefrontal cortex (PFC), consciousness, spike sorting, neural codes, bursts, alpha rhythm

## Abstract

This paper addresses two fundamental questions: (1) Is it possible to develop mathematical neural network models which can explain and replicate the way in which higher-order capabilities like intelligence, consciousness, optimization, and prediction emerge from the process of learning (Werbos, 1994, 2016a; National Science Foundation, 2008)? and (2) How can we use and test such models in a practical way, to track, to analyze and to model high-frequency (≥ 500 hz) many-channel data from recording the brain, just as econometrics sometimes uses models grounded in the theory of efficient markets to track real-world time-series data (Werbos, 1990)? This paper first reviews some of the prior work addressing question (1), and then reports new work performed in MATLAB analyzing spike-sorted and burst-sorted data on the prefrontal cortex from the Buzsaki lab (Fujisawa et al., 2008, 2015) which is consistent with a regular clock cycle of about 153.4 ms and with regular alternation between a forward pass of network calculations and a backwards pass, as in the general form of the backpropagation algorithm which one of us first developed in the period 1968–1974 (Werbos, 1994, 2006; Anderson and Rosenfeld, 1998). In business and finance, it is well known that adjustments for cycles of the year are essential to accurate prediction of time-series data (Box and Jenkins, 1970); in a similar way, methods for identifying and using regular clock cycles offer large new opportunities in neural time-series analysis. This paper demonstrates a few initial footprints on the large “continent” of this type of neural time-series analysis, and discusses a few of the many further possibilities opened up by this new approach to “decoding” the neural code (Heller et al., 1995).

## Alternate neural network models to explain/replicate consciousness (question 1)

Mathematical neural network models actually fall into two categories: (1) models of mature (fixed) neural circuits, such as elaborate models by Grossberg articulating what was learned by neuroscientists like Van Essen in deciphering specific visual pathways as they appear in visual cortex of a mature adult; (2) models of the more fundamental and universal learning capabilities of the brain, which aim to replicate competence in vision, decision-making, prediction, and other tasks as the emergent outcome of the learning process. This paper focuses exclusively on the second type of neural network model. That type of neural network model is itself a very large and diverse set. There have been efforts to combine the two types of neural network modeling (as in some efforts by Grossberg), but those are beyond the scope of this paper.

The effort to develop mathematical neural network models of intelligence and learning started from the seminal work of two groups: (1) the “cyberneticians” (Rav, 2002), such as Von Neumann, Wiener, and McCulloch, who developed the concept of neural networks as an approach to artificial intelligence; and (2) Donald Hebb, the neuropsychologist, whose book (Hebb, 1949) served as a manifesto to the new field of neural networks. Before Hebb, efforts to understand the dynamics of the cerebral cortex usually focused on very specialized attempts to understand the different functions of different Broca areas, in typical mature brains. Hebb called us to pay more attention to the experiments by Lashley on “mass action,” showing how any one area of the cortex can take over functions which are usually found in another area, when the latter is destroyed and when the required connections still exist. Walter Freeman, one of the important followers of Lashley, played a pivotal role in expanding our understanding of mass action in the brain (Freeman, 1975/2004); Karl Pribram and Jerry Lettvin, among others, also performed important experiments on that topic. In effect, Hebb challenged us to try to answer question (1) above, and many of us have tried to rise to this challenge.

In his final great work, Walter Freeman (with Robert Kozma) challenged a group of experimental neuroscientists and relevant theorists to submit chapters to a book addressing a key question (Kozma and Freeman, 2016): are the mathematical models now used in computational neuroscience powerful enough to answer question (1), and, if not, what changes are needed?

As part of that book, Freeman and Kozma ask whether neural network models would have to be extended, to account for field effects over three dimensions or even over quantum mechanical effects (Werbos and Dolmatova, 2016), in order to explain or construct the highest levels of intelligence or consciousness. Even if we focus for now on trying to understand the level of general intelligence which we see in individual brains of mice or rats (Werbos, 2014), it is possible that field effects *within* neurons give them a level of computational power beyond what traditional neural network models allow (National Science Foundation, 2008). Those extensions are important topics for research, but this paper will focus on simpler extensions, already an important part of the neural network field.

In the 1960's, neural network models inspired by digital computers (Rav, 2002) generally assumed that the brain itself must be like a digital computer, and hence that the “neural code” would consist of ones and zeroes, encoded simply as the presence or absence of spikes. Even today, many of the models used in computational neuroscience continue that tradition, by assuming that the neural code consists of spikes or pulses propagating and integrated in an asynchronous way, without any kind of master clock of the sort one would find in a modern computer.

Unfortunately, it was very difficult to find learning models capable of training such networks to perform even very simple tasks, let alone the more complex tasks which mammal brains can handle (Minsky and Papert, 1969). The neural network field languished and became even disreputable within artificial intelligence and engineering, until the field learned to accept a new type of learning model which required a different kind of neural code. The simplest version of the new type of learning model was renamed “backpropagation” (originally the name of a different algorithm by Rosenblatt), and simplified and popularized very widely (Rumelhart et al., 1986). Backpropagation involved two major new elements: (1) use of a continuous-variable neural code, instead of 1's and 0's; (2) use in learning of the derivatives of some error measure, calculated by signals propagating backwards in the network, with or without scaling enroute, justified by the general chain rule for ordered derivatives proven in 1974 for feedforward networks with or without time-delayed recurrence (Werbos, 1994) and generalized in 1980 to all types of recurrent network (Werbos, 2006). This kind of adaptation requires alternating cycles of forward calculation and backward calculation, which in turn requires a kind of master clock.

When this concept was presented to Minsky himself circa 1970, he objected that people in the modeling field know that there are no clocks in the brain, and know that all neurons use a code which is strictly binary, strictly defined by presence or absence of a spike. In reply, he was shown patch clamp recordings from higher centers of the brain, taken from Rosenblith, which demonstrated a sequence of volleys or bursts (Rosenblith, 1961) with regular timing; the volleys can be viewed as a set of spikes “on top of each other,” but the overall intensity of the bursts varied in a continuous way, from small bursts to large bursts. Thus, instead of viewing the data as a sequence of spikes at different times, one would view them as a continuous measure of intensity x_k_(t) for neuron k, where t takes on discrete integer values, relative to some kind of system-wide clock. Bursts continue to appear in the output of giant pyramid cells (Bear et al., 2007), cells which serve as the backbone and final output path of all parts of the cerebral cortex.

The rebirth of neural networks in the 1980s was based primarily on backpropagation, on learning models which assume a continuous neural code and an alternation of a forward pass to do computational work and a backwards pass for effective learning in the face of complex tasks. Also very important was a third class of neural network model (Grossberg, 1971), which we think of as the ODE type (ordinary differential equations), assuming a continuous neural code but, like the spiking models, asynchronous, and defined over continuous time.

It should be emphasized that the original, general form of “backpropagation” is a learning algorithm or a stream of local calculations implementing that algorithm. It is not the specific type of neural network topology, the Multilayer Perceptron (MLP), which was used most often in popularized books and simple applications. Backwards flows of calculation are needed for the efficient calculation of derivatives in general, whether scaled and modulated or unscaled (Werbos, 2006). The most powerful computational methods suitable for complex, general nonlinear tasks do require the calculation and use of derivatives. The topology proposed in our theory (Werbos, 2009) is more complex and powerful than the simple MLP.

This gives us three general families of neural network learning model in use today: (1) spiking; (2) the backpropagation family as defined here; and (3) ODE. Spiking and ODE models are very popular in computational neuroscience, and have been used in the analysis of real-time data from brains. Models of the backpropagation family have been much more widespread in engineering and computer science, where they have led to major breakthroughs in intelligent control (Lewis and Derong, 2012; Werbos, 2014) and in pattern recognition with “deep learning” (National Science Foundation, 2008; Ng et al, 2008; Schmidhuber, 2015). The main purpose of this paper is to discuss and illustrate how models in the backpropagation family can also be engaged and tested on multielectrode array data, and to show that the data available so far do not rule them out.

In this paper, we do not argue that models of the backpropagation family are sufficient to answer question (1), or to replicate the full range of higher-level capabilities we see in the brains of rodents. Rather, we would envision a kind of hybrid model, in which giant pyramid cells of neocortex receive clock pulses from the nonspecific thalamus at a key junction on the apical dendrite (Werbos, 2009), and output bursts under the control of that clock, while a complex network of interneurons provide Supplementary capabilities like associative memory, influenced by their inputs from the pyramid cells but not directly governed by a global clock. This is part of a more general theory of intelligence in the mammal brain (Werbos, 2009), grounded in general mathematical principles derived from analyzing what is required to achieve functional brain-like capabilities in tasks like decision-making and prediction of the environment (Werbos, 2010).

Many modelers correctly observed years ago that models based on the simplified popularized versions of backpropagation (like the MLP) would not be plausible as models of biological neural networks (BNN). However, deeper work on systems neuroscience has already revealed flows of information and types of synaptic connection supporting the idea that backward passes (as in the more general family of backpropagation designs) do exist in the brain (Smirnova et al., 1993; Buzsáki et al., 2012). A thorough review of learning and rhythms in the hippocampus (Kahana et al., 2001) shows that the mechanisms of learning do appear to vary as a function of the time of stimuli within the theta clock cycle, even though the origins of the theta clock in the hippocampus remain controversial. This paper focuses on the cerebral cortex, in part because the fibers from the nonspecific thalamus to the apical dendrites of giant pyramid cells have been well-established for decades, and in part because of the intrinsic importance of the cerebral cortex. It would be possible to model the oscillations in the nonspecific thalamus with ODE, but it is not really necessary at this stage, because they are so regular, and because they are essentially a hard-wired feature of the brain, not the kind of feature which emerges in detail from learning.

There is also an important connection between the theory of brain functioning presented in (Werbos, 2009) and the “Global Workspace” theory of consciousness developed by Bernie Baars, one of the top leaders in consciousness research (Baars, 2016). Baars argues that the information in our “conscious awareness” is basically just the current image of reality reconstructed in the cerebral cortex, by the “working memory” mechanisms described in wet neuroscience work by Goldman-Rakic and Legothetis, among others. Those researchers have observed that recurrent neural networks, with the kind of reverberations necessary for short-term memory, play a central role in this kind of consciousness. From mathematical work on functional requirements and training of recurrent networks (White and Sofge, 1992), we understand that a different kind of recurrence and training is required in order to produce this kind of short-term memory or “nonlinear state estimation,” compared with the kinds required for longer-term associative memory or “settling down” in image processing. Neither we nor Baars would say that recurrence in the brain is only of the time-delayed kind, but clocks and backwards passes turn out to be necessary for that kind, and for hybrid systems which include that kind of capability.

More concretely, the theory in Werbos (2009) proposes that the global workspace can be represented as the vector R(t) made up of the final axon burst outputs R_k_(t) of giant pyramid neurons k at clock time t, and that the cortico-thalamic system learns to build up this filtered image of reality and to predict inputs from the specific thalamus X(t) by a robust variation of the Stochastic Encoder/Decoder/Predictor (SEDP) model (White and Sofge, 1992). Simplified special cases of that model (like the Ford software for Time-Lagged Recurrent Networks) have won many recent time-series prediction competitions, but of course we expect the brain to have more powerful functional capabilities which include but surpass the simple TLRN. This is simply one way to translate the Baars theory into something we can test in a more fine-grained way on real-time brain data.

This theory of cortical function can also be seen as a way of implementing Llinas' theory of the brain as a prediction system (Llinas and Roy, 2009). Llinas' earlier work demonstrating highly precise synchronized clocks in the motor system of the brain is also relevant to the approach (Sugihara et al., 1993). In conversation at a workshop organized by Karl Pribram, Nicolelis reported that their important work on the cortico-thalamic system (Nicolelis et al., 1995) showed how cells in the thalamus which were initially good advance predictors of their neighbors [cells in x(t)] would relearn this prediction ability after it was destroyed by a lesion.

Strictly speaking, the theory in Werbos (2009) asserts that giant pyramid cells are adapted based on backwards error signals which are the sum of signals based on prediction error in the cerebro-thalamic circuit and on signals based on error signals from the basal ganglia and the limbic system, reflecting additional ways in which the brain can assess the quality of the outputs produced by the cerebral cortex. It asserts that the limbic system implements some variant of reinforcement learning (Lewis and Derong, 2012) which requires a global clock cycle twice as long (θ) as the clock cycle (α) required for prediction. It does not specify what drives the theta rhythm in the limbic system, but it allows for the possibility that the primary clock is the alpha clock in the nonspecific thalamus and cortex, and that the theta rhythms are somehow synchronized with that one. In any case, this paper focuses more on the cerebral cortex.

## Selection of real-time multielectrode data to test for clock cycles and backwards passes

The new work reported in this paper was initially inspired by (unpublished) comments by Barry Richmond of NIH, enroute to a meeting at the Dana Foundation. In his data on the neural code, he said that he saw a regular alternation between a short quiet period (on the order of 10–20 ms), a kind of “normal window” of signals flowing in the usual expected direction from inputs to outputs, on the order of 40–50 ms, and then a puzzling backwards window of 40–50 ms in which information seemed to go in the opposite direction. “I am not sure what to make of that second window, but I would guess that it has something to do with adaptation, somehow.” Given the prior work on neural network modeling, reviewed in the previous section, we found this to be very exciting, but we were unable to obtain more details, other than Richmond's published papers. The goal of this new work was essentially to reconstruct the details, by use of new data sets.

The new theory of cortical dynamics does not require the presence of a quiet period, but Richmond's observation suggested that it should be there. If so, it would be an excellent starting point for looking for a forward pass and a backwards pass. Thus, the first stage of our work was to look for that kind of regular quiet period.

Initially, we scanned the real-time Ecog data collected by Walter Freeman (Heck et al, 2016) to see whether it could be a basis for identifying quiet periods. Unfortunately, because this was data on field potentials at the outer surface of the cortex, the times of zero potential reflected a cancelation of positive and negative inputs to the neurons, rather than low activity as such. It seemed logical to expect that the “quiet periods” are best defined as periods when the outputs of the cortical pyramid cells (either zero or bursts, a monotonic output) were near to zero. Thus, we looked for real-time data from deeper in the brain, where they would reflect spikes or bursts output by neurons. (Unfortunately, we did not have access at that time to the spike-sorted parts of the Freeman data).

Note that simple Fourier analysis or wavelet analysis would not be a proper way to look for such regular quiet periods, because the activity in the brain at times which are not quiet depends a great deal on inputs which vary as a function of the experience of the rat or the mouse, and would show oscillations related to that experience (Heck et al, 2016; Kozma et al., 2012). The new theory does not question the existence of such important oscillations and activity, but it does require new methods of analysis in order to track the specific type of hardwired clock assumed here.

The next step was to thank Professor Jennie Si of Arizona State University for access to her extracellular data collected from deep in the brains of experimental rats (Yuan et al., 2015), data collected under NSF funding under a data management plan which promised public access to the data. Si warned us, however, that her real-time 16-channel data collected at 24 khz leaves open important and difficult questions about how to do spike sorting. In fact, when we looked for regular quiet periods in her raw data, we did not really find it. We found a mix of positive and negative signs as overwhelming as what we saw in the raw data from Freeman. There were a few hints of regular timing in plots in Excel of the high-pass filtered version of her data, at times of maximum activity in her experiments, but we decided to look for more monotonic data, more representative of the actual outputs of neurons, based on the current best state of the art in spike sorting, which we then studied in some detail (Harris et al., 2000; Buzsáki et al., 2012; Rossant et al., 2016).

All of the work reported here was performed using the database pfc-2 (Fujisawa et al., 2008, 2015) taken from the repository at crncs.org. All but some test and exploratory runs were based on two versions of the MATLAB file EE188_example, kindly emailed to us by Prof. Fujisawa of Riken. One version, about 3 megabytes in size, was identical to the file discussed in Fujisawa et al. (2008), underlying all its major reported results and Figures concerning local circuits and learning in prefrontal cortex. An expanded version, about 5 megabytes, included spike sorted data from an additional 32-channel silicon probe inserted into the CA1 region of hippocampus, from which real-time data were also collected on the same time scale (20 khz) in the same long sequence of sessions.

One of the great benefits of the pfc-2 database is that it includes a sorting of the pfc-neurons into three groups—confirmed pyramid cells, confirmed interneurons and confirmed others. This made it possible to estimate the location of quiet periods (start and end of each clock cycle) based on the spike sorted data from the pyramid cells only, and then use those estimates to analyze data from all of the pfc neurons. Because data was also available from CA1, we also performed a few analyses of CA1 data, but for reasons of time we used this data only for phase one of this work, the initial exploration of possible clock cycles. The greatest part of this work involved developing practical nonparametric analysis methods, and debugging and testing their use in MATLAB and in Octave.

The main part of these MATLAB files was a collection of six variables, of which we only used two:

(1) spiket(j), which gives the clock time at which spike number j was detected; and (2) spikeind(j), which contains a numerical ID (in the range from 1 to 400) for the neuron at which spike number j occurred. Of course, we also used the file containing the table of neuron types, identifying which was a pyramid cell and which was something else. We also used Excel to inspect the original spike sorted files, like EE188.res.1 and clu and fet, which contained the original version of the spike sorted data, and verified the simple exact mapping from those raw files to the more compact MATLAB files. Using that correspondence, it would be possible to repeat this analysis for all the sessions in the pfc-2 database, and evaluate the stability of the clock time over time, and with respect to sleep and wake states.

These two MATLAB files represented the best data we could find on the actual outputs of cortical neurons at the present time. However, the spike-sorting which was used to generate this data was all based on the concept of spike-based neural networks. It is only natural that the computational work on spike sorting has largely been inspired by the neural network models which are currently most popular, but this leaves open many questions about how well the spike-sorted data represents the actual output of the neurons, and about how to test models in which pyramid cells output bursts more than spikes. From the review in Harris et al. (2000), it is clear that regular behavior in brains may be more visible when we focus on bursts rather than spikes, but the full methodology of burst-sorting given in Harris et al. (2000) was beyond the time and resources available for this initial work. In consequence, we used a very simple routine for burst-sorting, based on the easy half of the procedure described in Harris et al. (2000): we filtered all the spikes in the MATLAB file down to a smaller file, in which we simply threw out all spikes which were not accompanied by other spikes from the same neuron within 6 ms of the same time. We found that the burst-filtered version of the pfc2 database was only about 1/3 less than the size of the original database, and that all of the measures of pattern which we looked for were stronger on the burst-filtered version of the database.

We did consider trying to use the fet files in the pfc-2 database to perform the additional filtering used in Harris et al. (2000). However, some of the features in that file seemed to call for the use of distance measures based on distance, while others appeared to be more like measures of intensity, calling for the use of measures like inner product. Clearly it will be an important research task for the future to sort out these kinds of issues, to organize the development of burst sorting and measurement in a more systematic way, and to apply them to databases like pfc-2.

## Computational methods and results used to probe for clock cycles and direction of signal flow

Effective and robust dynamic modeling of complex systems like the cerebral cortex generally requires that we start with a phase of exploratory data analysis, in order to avoid missing major patterns and being limited by initial assumptions (Hoaglin et al., 1983). The measures used here were developed in order to be as simple and direct as possible, for this early stage, while—most importantly—articulating or estimating the key hypotheses under study (Werbos, 1990). The issue of robustness is very tricky, in a situation where the raw data includes only about 100 variables which are part of a very information rich highly nonlinear system containing billions of neurons evolved over millions of years to handle the maximum throughput of information (Macke et al., 2011). We strongly hope that future research will probe these theoretical issues in more detail. Here we will simply report what the exploratory measures are which we used, and leave the refinements to the future.

**Figure 1:**
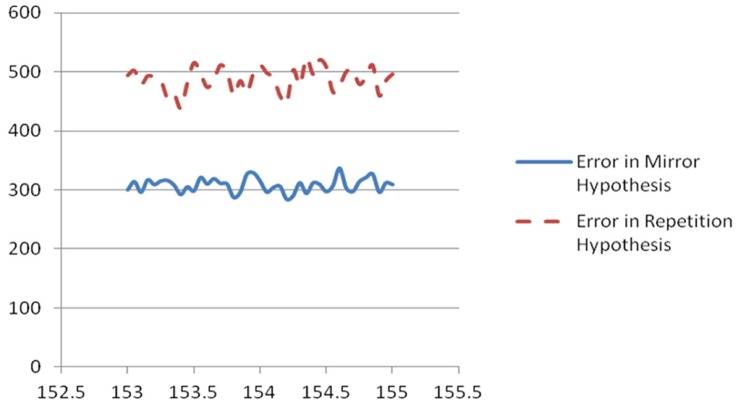
**Plot of scaled unweighted e↓ and e↑ vs. assumed clock time from Table 2**.

We developed and used four sets of computational measures here. All four required us (the user) to specify a candidate clock cycle time, and another parameter K, to be discussed further below. All four call out for some combination of simulation studies (like those used in Werbos, 1994) to assess the robustness of competing statistical methods) and mathematical analysis to develop more formal measures of statistical significance—though the main results in **Table 2** and Figure 1 are large enough to be clear already.

### Summary of methods and findings

For phase one of this work, we developed “quiet time” measures, to tell us whether there exists an interval of 10 ms, at the same phase of every clock cycle, over K clock cycles, which regularly experiences fewer spikes than other times in that clock cycle. We applied these measures to the four most active neurons in the entire database individually, to the pyramidal cells, and to larger sets of neurons, with or without burst filtering, for all possible clock times which were integer multiples of 0.1 ms between 100 and 200 ms. The four most active neurons included three cells in the hippocampus (neurons number 329, 349, and 373), and an interneuron in the cerebral cortex (120). The interneuron and neuron 349 did not show regular quiet periods, but neurons 329, 373 and the pyramidal cells in prefrontal cortex as a group all showed regular quiet periods, at similar ranges of time intervals, with *K* = 100, *K* = 1000, and even *K* = 10,000. The strongest range of possible clock cycle times (the range with the quietest quiet periods) was 154 ± 1 ms, but 145 ± 1 and 134 ± 2 seemed plausible enough to warrant further investigation. It was striking that the same clock periods seemed to be best for all three data sets, with all three choices for K. However, it was also disappointing that we could not be sure what the best estimate of clock time would be, within those ranges, for the available data using this measure.

In phases two and three of this work, we mainly focused on using the quiet time results to identify clock cycles, and to test whether the sequence of firing in the later half of a clock cycle (“PM”) is more like a repetition of the sequence in the first half (“AM”) or like a reversed sequence, or mirror image. We also hoped that further analysis would give us more accuracy and certainty in knowing what the clock time is; that hope worked out for phase three, but not for the simpler work in phase two.

Before starting this work, we dreamed of studying neurons arranged in a network, such that we could actually see the “lights” (firing) moving from back to front in a forward pass (“AM”) and then from front back in a backwards pass (“PM”). However, spike sorting provided only neuron IDs, not physical location. Fujisawa et al. (2008) provided what may be the best identification of neural networks from spike sorting available in the literature, but the identification did not cover most of the neurons in the dataset, and it was based on a cross-correlogram methodology which raises questions about robustness and possible systematic error. Thus, for phase two we looked at simple measures which describe the sequence of firing of individual neurons within a clock cycle, while for phase three we looked at which neurons fire in what order or sequence. The phase three results look more interesting, but for completeness we will also describe the phase two results. Both in phase two and phase three, the error in assuming mirror-image signal propagation was about 1/3 less than the error in assuming repetition of the same sequence, across all candidate clock cycle times, and K of 20, 100, and 1000 (the three choices we considered). In retrospect, we suspect that there might also be a useful way to exploit the information in the pfc-2 database about which neuron belongs to which of the twelve shanks, which does give some information about locations.

In phase 3, we also calculated three measures of “inertia,” of the tendency for the same list of neurons to fire from one clock cycle to the next, in the same sequence in AM or PM. This generally sharpened and validated our estimate that 153.4 ms is the correct clock cycle time all across this data (session 188 for the rat identified as EE). We looked a bit for evidence of phase drift or cycle time drift from one span of data to another (where a “span” is K time cycles), but did not find any within this session.

### Details of phase one methods and results and continued quiet time analysis

For phase one, we developed and debugged a sequence of MATLAB functions to input recorded data from the brain, and report back how quiet the quietest phase of the proposed clock cycle time was. More precisely, we ultimately developed a function, Find_clock_in_spiket, for which the user would supply three input arguments: (1) delta, an integer, the proposed clock cycle time in the same units of time assumed in the spiket data; (2) K, number of clock cycles per span of data to be analyzed; and (3) spiket(j), an array simply containing the time at which a spike was observed, for all spikes j recorded (in order) in the dataset being analyzed. We also developed a simpler variation, find_clock_in_power, to analyze data of the form xpower(t), representing the time series of a nonnegative measure of signal power, tested on the Si data (Yuan et al., 2015).

To visualize the algorithm and the mathematical issues, it may help to consider a clock cycle of the brain by analogy to the 24-h cycle of a clock. If *K* = 100, we organize the spike data into the hours of 100 days. We calculate a histogram of what time of day the spikes occurred, in each span of 100 days. If activity was quietest, say, between 2 P.M. and 3 P.M. across all 100 days, then we measure “quiet power” for that interval as the sum of activity during that hour, summed over all days in the span, and we compare that later to the average activity across all hours. (Instead of an hour here, we actually looked for a quiet 10 ms interval, and considered all possible intervals starting from the beginning of the cycle, starting from 2 ms after the start of a cycle, and so on). The overall quiet time score for the entire dataset is simply the sum of the quiet time scores for each span of data in that dataset. (The function calculates the number of spans simply as the length of times in the database divided by the time length per span. The length of times in the database is simply the highest and last value of spiket(j), minus the starting time, spiket(1). “Left over” spikes, beyond the last whole span of time, are simply not used in the analysis. The final version of this function handled left-over spikes in a cleaner manner, and changed a few numbers slightly, but all qualitative results were similar to those with the early versions).

Now: what would happen if one applied such an algorithm with the wrong measure of the length of a day? For example, if one were to aggregate hourly electricity consumption data assuming a 25-h day, after about 30 “days” one would expect the measurements to be hopelessly out of synchronization, and the histograms would be flat. For this reason, we initially hoped that use of the quiet time measure would give a very sharp indication of what the clock time is, for those cells which actually are governed by a very regular clock. This would allow us to analyze issues like forward vs. backward signal propagation, in phases two and three, by simply using the precise clock estimates from phase one. A fuzzy estimate of clock time reduces the accuracy of any phase two and phase three analyses which depend on them.

However, the quiet time analysis by itself proved useful only as a screening method. As discussed in Section Summary of methods and findings, it identified four reasonable ranges of possible clock time, each 2 ms wide. Thus, for phases 2 and 3, we performed more intensive analysis of all possible clock times in those four ranges, for all multiples of 0.05 ms, measuring not only quiet power but additional measures. Table 1 illustrates the final results of phase one, giving the quiet power (number of spikes across proposed quiet periods) for 21 possible choices of clock cycle time in the most promising of the four ranges considered, for the choices *K* = 1000 and *K* = 10,000. Notice that the row of Table 1 next to the bottom gives the total number of spikes counted in each analysis, and the bottom line gives the average number of spikes one would expect in a random 10 ms interval.

**Table 1:** **Quiet power vs. possible clock time in milliseconds**.

***K* = 1000**				***K* = 10000**		
**Proposed clock time in ms**	**Neuron 329**	**Neuron 373**	**All pyramids in pfc**	**Neuron 329**	**Neuron 373**	**All pyramids in pfc**
153	4111	1038	141	3160	711	149
153.1	4225	991	133	3215	740	158
153.2	4231	983	145	3254	732	149
153.3	4398	1067	141	3340	762	165
153.4	4452	999	127	3307	741	147
153.5	4437	1007	143	3351	747	149
153.6	4507	1036	150	3372	753	155
153.7	4527	1031	154	3320	765	144
153.8	4551	1067	136	3421	751	159
153.9	4666	1072	129	3423	745	137
154	5323	1142	137	3404	754	149
154.1	4755	1084	143	2831	648	141
154.2	4953	1076	131	2934	695	155
154.3	4898	1101	135	2922	653	141
154.4	4938	1070	159	2911	615	141
154.5	5096	1128	127	2987	704	141
154.6	5059	1100	129	3056	697	137
154.7	5069	1096	140	2975	701	157
154.8	5182	1118	133	3146	716	159
154.9	5121	1129	145	3146	714	149
155	4145	983	153	3176	738	159
Nspikes	92117	22432	4820	54658	12524	2908
Null	5981.623	1456.623	312.987	3549.221	813.2468	188.8312

Note that the spikes in the quiet time are quite a bit less than what one would expect in a random “hour of the day,” most notably for clock times of 153.4 and 154.5 ms, with *K* = 1000. The actual score of 127 is much less than the null expected score of 313. If the true clock time were, say, 153.35 ms, after 1000 cycles, one would expect missynchronization of 0.05^*^1000 = 50 ms from the start of the span to the end; thus, when we only know the clock time to within 0.05 ms, it is quite remarkable to have such a degree of quiet power with K as high as 1000. (It is possible only because the actual quiet time interval may be a bit wider than 10 ms, as Richmond initially suggested, and because a *K* = 1000 implies that the cycles considered within each span are not more than 500 cycles away from the middle of the span). On the other hand, it is clear that these results do not tell us very clearly what the best candidate time is within this 2 ms window. Quiet power was of course higher, relative to the null expectation, in other time ranges.

The MATLAB function Find_quiet_time_in_spiket reports out the quiet power as described above, the total number of spikes actually considered in each quiet time analysis, and the number of complete spans found in the data. (If the user proposes a K too large for the dataset, the function was designed to consider the entire database as one span; however, we never actually tested that feature). It also provides an output array, wherebin, which can be used in debugging, in analysis, and in support for other MATLAB functions as in phase 2 and phase 3. For each of the spans identified in the data, it tells us “at what hour” the quietest period was, how many spikes were found in quiet periods in that span, and how many spikes in total were counted in that span. For example, with *K* = 10000, we only had seven spans in the EE188 data! If the location of the quiet period drifted systematically up or down in the wherebin data, this would suggest that a more refined estimate of the clock time would improve results; however, in our initial exploration of those diagnostics, we found no indications of such systematic drift. Note that we used underbars in the names of all of our MATLAB functions, simply because of how MATLAB works.

Finally, we note that the arithmetic of this analysis was simplified by the fact that the “spiket” variable in the pfc-2 database was based on a recording rate of 20,000 measurements per second (Fujisawa et al., 2008), such that the allowed values of “delta” represented multiples of 0.05 ms. It would have been possible to consider clock times with even more temporal detail, simply by multiplying the entire array “spiket” by 10, so that any clock time which is a multiple of 0.005 ms could be evaluated. That is one of the many variations and extensions which could be considered in future work.

Another extension would be to study whether the clock cycle time is or is not the same for the rat called EE in all the different sessions recorded in the original data (Fujisawa et al., 2008, 2015). The underlying theory [Section Alternate neural network models to explain/replicate consciousness (question 1)] suggests that it might be, but experiments with other large shifts in global brain parameters (as with hormones or alcohol) suggest that brains may be able to learn to be robust with respect to them, and hence that natural selection may have permitted them.

### Phase 2 methods, analysis and results

Phase 2 and phase 3 both addressed the question: is the real-time data available here consistent with what Richmond said about alternating forward and backward passes in the cortex, as the backpropagation family of neural network models would predict?

The backpropagation family of models does not predict that the sequence of firing in the backwards pass is a perfect mirror image of the sequence in the forwards pass, with a cycle of brain operation. It may be more or less of a mirror image, depending on the degree of fast recurrence in the interneurons, the impact of long-term memories, and the structure of the tasks currently faced by the organism; the importance of current tasks in bringing out different aspects of network structure is illustrated very vividly in (Fujisawa et al., 2008). Nevertheless, the backpropagation models would predict that the backwards pass looks more like a mirror image of the forward task than like a repetition of the forward pass. If calculations were always running forward, both in the first half of a brain cycle and in the second half, one would expect the opposite result: the second half would look more like a repetition than like a mirror image.

The main goal of phases 2 and 3 was to find out which of these two possibilities better fits the data. Phase 2 took a minimal approach, trying to compare the two hypotheses (mirror vs. repetition) without making any assumptions at all about the relations and connections between different neurons. Phase 3 made use of the neuron ID information, and in our view, is much more conclusive and robust. Nevertheless, both types of measure strongly favored the mirror hypothesis over the repetition hypothesis.

Of course, to compare the events in the first half of a brain clock cycle with those in the second half, one must identify the time interval for all of the brain cycles to be analyzed. The phase 2 analysis was performed by a MATLAB function, Test_hypotheses, which started out by calling Find_clock_in_spiket (discussed in Section Details of phase one methods and results and continued quiet time analysis above) to identify the quiet intervals (10 ms wide) in each of the formal clock cycles.

The formal clock cycles which Find_clock_in_spiket starts from are different from the actual brain cycles it locates. In any span of data, Find_clock_in_spiket analyzes K intervals of time, formal clock cycles, and it calculates where the quiet interval is relative to the start of the formal cycle. In Test_hypotheses, a brain cycle is defined as the interval of time stretching from the middle of the quiet period in one formal cycle, to the next quiet period in the next formal cycle. Since there are K formal clock cycles in any span of data, this yields K-1 brain cycles. For each brain cycle, we may define t_−_ as the start time of the cycle, t_+_ as the end time of the cycle, and t_0_ as the exact mid-point between the two. We define the “AM” period of the brain cycle as the interval between t_−_ and t_0_. We define the “PM” period as the interval between t_0_ and t_+_. Both in phase 2 and in phase 3, our goal was to answer the question: “Is the sequence of neurons firing in the PM period more like a mirror image of the sequence in the AM period, or like a repetition, over the entire dataset?”

In phase 2, we calculated two measures of error for each of the two hypotheses (mirror vs. repetition), for every neuron which fired at least once both in the AM part of the brain cycle and in the PM part of the brain cycle. We calculated these four measures for each of the identified brain cycles, and simply added up total error over all brain cycles to generate the final error scores. We weighted the error by the number of spikes, because this reflects the greater importance of neurons and times of greater activity. As in phase one, we used the burst-filtered data from the pyramid cells to establish the clock intervals, but all of the remaining analysis used the entire burst-filtered dataset of all recorded neurons in the prefrontal cortex.

For each active neuron, in each clock cycle, we began by find out t_AM_^+^, the time of the last spike in the AM period, and t_AM_^−^, the time of the earliest spike in the AM period, and then t_PM_^−^ and t_PM_^+^. We also calculated N, the total number of spikes for that neuron in the AM and PM periods.

The first two measures of error tested whether the interval between first and last spike for that neuron in the PM matches a mirror image (through t_0_) of the first and last spike in the AM, or whether it matches a repetition. We calculated the error in the mirror hypothesis as:

(1) $$\text{e}↓={\text{N}}^{*}(|({\text{t}}_{0}-{\text{t}}_{\text{AM}}{}^{+})-({\text{t}}_{\text{PM}}{}^{-}-{\text{t}}_{0})|+|({\text{t}}_{0}-{\text{t}}_{\text{AM}}{}^{-})-({\text{t}}_{\text{PM}}{}^{+}-{\text{t}}_{0})|)$$

We calculated the error in the repetition hypothesis as:

(2) $$\text{e}↑={\text{N}}^{*}(|({\text{t}}_{0}-{\text{t}}_{\text{AM}}{}^{+})-({\text{t}}_{+}-{\text{t}}_{\text{PM}}{}^{+})|+|({\text{t}}_{0}-{\text{t}}_{\text{AM}}{}^{-})-({\text{t}}_{+}-{\text{t}}_{\text{PM}}{}^{-})|)$$

The next two measures were essentially the same, except that we compared the midpoints of the AM and PM intervals.

We ran the Test_hypotheses function using all four of the intervals discussed above for the possible clock time, with *K* = 1000. For the most plausible interval, from 153 to 155 ms, we also tried *K* = 100. We divided the error scores by 1,000,000 and rounded to the nearest integer, so as to provide a nice table, for each of the 41 clock times considered in each interval, giving a new estimate of quiet power, and results on each of the four error measures.

For the interval between 153 and 155 ms, every choice of clock cycle gave a score of 27 or 28 for e↓, and every choice of clock cycle gave a score of 36,37 or 38 for e↑, with *K* = 100 and with *K* = 1000. In other words, the mirror hypothesis was notably preferred over the repetition hypothesis for all choices of clock time and both choices of K. The mirror hypothesis was also preferred in a uniform way in the other three intervals: for 134–136 ms, e↓ was 23 or 24, vs. e↑ of 31, 32, or 33; for 144–146 ms, it was 25,26, or 27 vs. 33, 34, or 35; for 132–134 ms, it was 23 or 24 vs. 31 or 32. Likewise, for the midpoint error measure, in the 153–155 ms interval, it was 12 vs. 17 (with only two cases of 16 and 17). The corresponding quiet time error with *K* = 1000 was 134 at 153.4 ms, lower than the quiet time error at other candidates in that interval, and notably lower than the quiet time error in any of the other three intervals.

In summary, the phase two measures of hypothesis error did favor the mirror hypothesis over the repetition hypothesis, for all choices of possible clock time. 153.4 ms emerged a bit more clearly as the best estimate of the underlying clock time. We believe that the results from phase three are more robust and more convincing than those of phase two, but it is even more notable that two entirely different ways of evaluating the clock time and the mirror hypothesis led to the same conclusions.

### Phase 3 methods and results

The phase 3 analysis was performed using a MATLAB function, Test_sequence_and_inertia, which provides three sets of statistical measures for each user-supplied choice of clock cycle time and K. First, it calls Find_clock_in_spiket, exactly as Test_hypotheses does, and outputs a quiet power measure, exactly the same as the quiet time measure calculated by Test_hypotheses. It also provides four new measures of error for the mirror hypothesis and the repetition hypothesis. Finally, it provides three useful measures of inertia or autocorrelation, which provide another way to evaluate whether the proposed clock cycle time is the correct one.

As in phase two, the four measures of error are calculated for each brain cycle, and added across all brain cycles and scaled, to get the total measures of error for the mirror hypothesis e↓ and for the repetition hypothesis e↑. Within each brain cycle, we first create a list of active neurons—neurons which fired both in the AM and in the PM. If there was only one active neuron, or none, this brain cycle is skipped, because there are no AM and PM sequences to be compared. Next, for each active neuron, we calculate the two simple averages, (t_AM_−+ t_AM_+)/2 and (t_PM_−+ t_PM_+)/2, which indicate when this neuron fired, both in AM and PM. We sort the neurons according to when they fired in the AM and when they fired in the PM. The unweighted measure used for e↑ is simply the inversion number comparing these two permutations; the inversion number (Foata, 1968) is a widely used standard measure for comparing the similarity of two permutations. The unweighted measure used for e↓ is the inversion number comparing the AM sequence and the reverse of the PM sequence. The weighted versions in each brain cycle are equal to the unweighted versions multiplied by the product of the total number of spikes in all active neurons in the AM and the total number in the PM.

The results of this analysis for the most important case are shown in Table 2 and in Figure 1.

**Table 2:** **Phase 3 results for 153–155 ms, with *K* = 1000**.

**Clock time in ms**	**Measures of clock accuracy**				**Measures of mirror vs. repetition**			
	**Quiet power**	**Change of neurons**	**Sequence change**		**Unweighted**		**Weighted**	
			**AM**	**PM**	**e↓**	**e↑**	**e↓**	**e↑**
153	151	3689	13	11	300	494	20463	29868
153.05	149	3699	19	21	314	502	23197	32001
153.1	141	3671	20	19	296	479	20355	30066
153.15	154	3709	19	11	317	493	21875	28649
153.2	153	3669	19	21	309	491	21129	30163
153.25	135	3590	24	19	315	486	22258	31344
153.3	150	3643	25	18	316	456	22863	28318
153.35	136	3530	19	19	308	463	21739	30052
**153.4**	**134**	**3620**	**11**	**17**	**292**	**439**	**21967**	**27835**
153.45	137	3650	18	14	305	481	22080	30627
153.5	152	3659	16	22	298	515	23334	30109
153.55	143	3628	20	22	321	497	23171	31574
153.6	160	3611	23	20	310	474	23090	28241
153.65	140	3625	26	25	319	487	22364	29398
153.7	164	3673	22	26	311	511	22154	30875
153.75	154	3610	20	22	310	501	22823	30298
153.8	146	3573	14	19	287	461	21342	29518
153.85	153	3648	17	13	296	485	21126	32104
153.9	142	3541	18	19	327	465	23432	28995
153.95	150	3659	17	19	329	498	23760	31858
154	150	3659	22	20	315	511	22280	32133
154.05	143	3620	20	10	296	498	23683	30275
154.1	152	3696	18	19	304	490	23563	29190
154.15	138	3660	23	19	306	460	23271	28663
154.2	141	3567	15	17	284	448	21117	28161
154.25	135	3624	22	19	290	503	22837	29861
154.3	144	3660	27	25	312	478	22404	31269
154.35	151	3603	28	24	294	523	21108	32955
154.4	168	3619	21	22	312	496	20953	32755
154.45	150	3660	19	16	309	520	23933	31771
154.5	135	3635	21	23	297	508	22120	33437
154.55	137	3646	19	17	307	466	21698	29341
154.6	142	3582	18	19	337	477	24138	29713
154.65	156	3597	23	22	303	500	22021	30999
154.7	149	3633	16	16	297	503	21210	32023
154.75	143	3625	20	17	314	479	22250	29081
154.8	142	3665	25	18	321	492	23359	31363
154.85	151	3676	20	18	327	511	25244	30280
154.9	155	3611	14	18	296	460	21002	30199
154.95	160	3639	18	20	312	485	22848	30045
155	159	3602	21	16	309	496	22264	33578

To understand the meaning of the e↓ and e↑ error measures, it may help consider two examples where 5 neurons (numbered N1–N5) fire in the following sequences within a clock cycle, and where t0 is the mid-point of the time cycle:

case A: N1, N2, N3, N4, N5, t0, N1, N2, N3, N4, N5

case B: N1, N2, N3, N4, N5, t0, N5, N4, N3, N2, N1

In case A, the unscaled value of e↑ is zero, because the sequence of firing after the mid-time is identical to the sequence before; however, the unscaled value of e↓ is 10 (4+3+2+1), because it takes 10 swaps to make the sequence after t0 match the mirror image of the sequence before. Case B is the opposite. The four columns on the right of Table 2 are all sums of e↓ or e↑, unweighted or weighted by the level of neuron activity in the time cycle, scaled by the same factor for convenience in printing.

The first of the three new inertia measures in Table 2 is simply the number of neurons which were added or dropped out from the list of active neurons, from one brain cycle in a span to the next. The second is the inversion number comparing the sequence of neurons firing in the AM, for those neurons which are active both in one brain cycle and the next. The third is the same, for the PM sequence.

## Discussion

Neural network models in the large family of backpropagation-based models have already performed well in challenging applications demanding an ability to replicate the kind of abilities brains have proven possible from pattern recognition to intelligent control, with a strong foundation in the technology disciplines which specialize in designs capable of addressing such tasks in a highly effective manner. There is every reason to believe that hybrid systems, effectively combing the capability of backpropagation networks and other types of network more common in computational neuroscience, could do still better in allowing us to replicate and understand the higher-order learning capabilities which drive mass action in the mammal brain, if we could make contact between the world of functional, mathematical neural network models and the world of empirical real-time data in neuroscience.

This work has done a quick initial evaluation of whether two key ideas in backpropagation might actually fit empirical real-time data from the brain, using a series of new quantitative measures which directly capture two of the most important predictions of that type of model—the prediction of a regular clock cycle, and of an alternation of forward and backward passes of calculation. We hope that these new measures inspire more work to address the many questions which flow from considering this new class of models of brain functioning.

A few of these questions and opportunities for future research were already discussed above, but there are many more. For example, it would be interesting to revisit the work of Fujisawa et al. (2008), and see what the cross-impacts and networks look like when the full database is partitioned into AM data and PM (and leftovers at the boundaries between spans). It would be interesting to revisit the work on models to predict neural signals over time, not only in burst-sorted and spike sorted data, but even in the original raw data, when we have the ability to partition that data into AM and PM, and to use the clock cycle here to use seasonal adjustment types of method as in time-series analysis (Box and Jenkins, 1970); see (Werbos, 1994) and (Werbos, 2010) for the extension of such time-series analysis methods to the multivariate and nonlinear cases, respectively. Because financial market data, like spike data, tend to involve discrete events and irregular kinds of statistics, it is quite possible that the approach used in (Werbos, 2010), drawing on Peters (1996), could yield new insights in this context. And of course, these MATLAB functions, developed to be very general in nature, could be applied to other databases.

The effort to understand the mathematical and computational principles underlying intelligence in the mammal brain is perhaps one of the two most important and fundamental challenges to all of basic science for the coming century. (The other is the continuing quest to understand the fundamental laws of physics). It is also a key motivation for society as a whole to be interested. It is hoped that this work will inspire new work which fully rises to that grand challenge.

A reviewer of this paper has raised an interesting question: can we try to imagine new classes of model, beyond those discussed in Section Alternate neural network models to explain/replicate consciousness (question 1), which would also be functional in information processing, but would treat time in a different way and fit our results from a very different basis? In fact, the work reported in Werbos and Dolmatova (2016); Werbos (2016a,b) does begin to suggest more radical types of model and technology which may or may not be relevant to understanding the basic rodent brain which we see in the laboratory.

Human cultures disagree violently at times about the nature of human consciousness, beyond the level of what we share with mice and rats. While we may hold different viewpoints (Werbos, 2012, 2015; Davis, 2016) on that larger question, beyond the reach of consensus science at present, we hope that we can agree that better understanding of what we share with mice and rats is an important steppingstone to understanding how to achieve the highest potential which we as humans can attain.

## Author contributions

All authors listed, have made substantial, direct and intellectual contribution to the work, and approved it for publication.

### Conflict of interest statement

The authors declare that the research was conducted in the absence of any commercial or financial relationships that could be construed as a potential conflict of interest.
